# Effectiveness of a Treatment Switch to Nevirapine plus Tenofovir and Emtricitabine (or Lamivudine) in Adults with HIV-1 Suppressed Viremia

**DOI:** 10.1371/journal.pone.0128131

**Published:** 2015-06-24

**Authors:** Josep M. Llibre, Isabel Bravo, Arelly Ornelas, José R. Santos, Jordi Puig, Raquel Martin-Iguacel, Roger Paredes, Bonaventura Clotet

**Affiliations:** 1 HIV Unit and "Lluita contra la SIDA" Foundation, University Hospital Germans Trias i Pujol, Badalona, Spain; 2 Universitat Autònoma de Barcelona, Barcelona, Spain; 3 Department of Econometrics, Statistics and Economy, University of Barcelona, Barcelona, Spain; 4 Infectious Diseases Dept, Odense University Hospital, Odense, Denmark; 5 Universitat de Vic (UVic). Vic, Catalonia, Spain; McGill University AIDS Centre, CANADA

## Abstract

**Background:**

Switching subjects with persistently undetectable HIV-1 viremia under antiretroviral treatment (ART) to once-daily tenofovir/emtricitabine (or lamivudine) + nevirapine is a cost-effective and well-tolerated strategy. However, the effectiveness of this approach has not been established.

**Methods:**

We performed a retrospective study evaluating the rates of treatment failure, virological failure (VF), and variables associated, in all subjects initiating this switch combination in our clinic since 2001. Analyses were performed by a modified intention to treat, where switch due to toxicity equalled failure. The main endpoint was plasma HIV-RNA < 50 copies/mL.

**Results:**

341 patients were treated for a median of 176 (57; 308) weeks. At week 48, 306 (89.7%) subjects had HIV-1 RNA <50 copies/mL, 10 (2.9%) experienced VF, and 25 (7.4%) discontinued the treatment due to toxicity. During the whole follow-up 23 (6.7%) individuals (17 on lamivudine, 6 on emtricitabine; p = 0.034) developed VF and treatment modification due to toxicity occurred in 36 (10.7%). Factors independently associated with VF in a multivariate analysis were: intravenous drug use (HR 1.51; 95%CI 1.12, 2.04), time with undetectable viral load before the switch (HR 0.98; 0.97, 0.99), number of prior NRTIs (HR 1.49; 1.15, 1.93) or NNRTIs (HR 3.22; 1.64, 6.25), and previous NVP (HR 1.54; 1.10, 2.17) or efavirenz (HR 5.76; 1.11, 29.87) unscheduled interruptions. VF was associated with emergence of usual nevirapine mutations (Y181C/I/D, K103N and V106A/I), M184V (n = 16; 12 with lamivudine vs. 4 with emtricitabine, p = 0.04), and K65R (n = 7).

**Conclusions:**

The rates of treatment failure at 48 weeks, or long-term toxicity or VF with this switch regimen are low and no unexpected mutations or patterns of mutations were selected in subjects with treatment failure.

## Introduction

Many subjects on suppressive antiretroviral therapy (ART) may be considered candidates for long-term regimen simplification towards easier to administer, more tolerable, or more cost-effective regimens [1–3]. Treatment guidelines consider that boosted protease inhibitors (PI) or efavirenz may be switched for toxicity, simplification, prevention or improvement of metabolic abnormalities or adherence facilitation to unboosted atazanavir, non-nucleoside reverse transcriptase inhibitors (NNRTIs; NVP, efavirenz, rilpivirine), or integrase inhibitors (raltegravir or elvitegravir/cobicistat) [1–7].

Nevirapine (NVP) displayed similar rates of efficacy at 12 and 36 months against efavirenz in simplification [8–13], and achieved the lowest rates of virological failure and higher lipid benefits in the extended three-year follow-up in a randomized study [12;14;15]. However, some observational cohorts found higher rates of virological failure with nevirapine versus efavirenz [16–18]. Baseline uncontrolled biasing factors, mainly differences in calendar year of prescription, could have an impact on the results of these cohorts. In addition, it is one of the antiretroviral drugs that achieve higher reductions of residual plasma viremia to below 1 copy/mL and a better lipid profile [15;19–21]. Pre-treated individuals with high CD4 cell counts do not have the increased risk for treatment-limiting toxicity seen in naives, provided there is no detectable viremia at initiation of NVP [22]. NVP has a well-known initial potential toxicity profile, and has not been associated to any specific long-term toxicity.

Among the newest drugs, only elvitegravir/cobicistat has been evaluated in randomized studies as a switch strategy for subjects receiving NVP [7]. It demonstrated non-inferior efficacy in the substitution of efavirenz or nevirapine, albeit the sole benefit (lipid profile) in the study was seen only in the efavirenz subgroup. Therefore, maintenance of generic NVP in long-term therapy might offer a powerful approach to cost-savings in well-resourced countries, and be a common strategy in countries with limited treatment options [23]. However, some patients and physicians may believe that a new brand-name drug is superior or more appealing, and could be reluctant to maintain an effective antiretroviral regimen based on a generic drug.

The combination of once-daily NVP plus TDF/FTC (or 3TC) has been extensively used as a long-term simplification regimen in some European countries, however information about the efficacy and long-term toxicity of this regimen is still scant [24;25]. Furthermore, 3TC has been associated with lower virological responses compared to FTC in some reports in naives, including one with NVP and TDF, but data are not available in simplification [26].

Therefore, accurate data on the long-term efficacy and toxicity of NVP plus TDF/FTC (or 3TC) as a switch regimen—with particular focus on the rates of VF or any particular pattern of unexpected mutations—are needed.

## Methods

### Study design and study subjects

We performed a retrospective cohort study of HIV-infected patients attending a tertiary University Hospital in Barcelona, Spain since 2001, when all drugs became available. All subjects aged ≥18 years with documented HIV-1 infection were included if they started treatment with NVP plus TDF plus FTC (or 3TC) as a switch from any previous regimen, with an undetectable plasma viral load (pVL), and had at least one subsequent follow-up visit. The inclusion criteria allowed incorporating subjects with early withdrawal of the regimen due to toxicity. Subjects were followed until they stopped the regimen for any reason. The study was approved by the Hospital Research Ethics Committee and conducted in accordance with the Declaration of Helsinki and National standards.

Historical follow-up data were extracted from medical records through a systematic database search. No restriction criteria were included in the search. All causes of treatment discontinuation were registered. VF was defined as two consecutive pVL >50 copies/ml.

Baseline characteristics were gathered, including age, gender, risk factor for HIV acquisition, time of HIV infection, number of prior antiretroviral drugs and antiretroviral regimens, prior NRTI mono or dual therapy, time with HIV-1 RNA suppression before the switch, hep B or C co-infection, and reason to initiate the study regimen. CD4^+^ cell counts and pVL were collected every 12–24 weeks thereafter, until the last sample available.

The complete previous treatment history was searched, and all previous NNRTI interruptions were recorded, as well as all prior treatment failures.

Genotypic resistance tests prior to the initiation of the regimen and the available resistance studies in those patients who failed were collected.

### Statistical analysis

Patients’ characteristics were described using medians (IQR) for continuous, non-normal variables and percentages for categorical variables.

The primary endpoint was the proportion of subjects with pVL <50 copies/mL at 48 weeks. We based our efficacy and safety analyses on a modified intent-to-treat (mITT, S = F) exposed or safety populations, which consisted of all patients initiating the regimen with any treatment discontinuation due to toxicity or voluntary treatment discontinuation considered as treatment failure. Subjects substituting 3TC with FTC during the study were not considered failures. Patients lost to follow-up or withdrawing the regimen due to reasons unrelated to toxicity (i.e. recruited into a clinical trial) or efficacy were censored at that time in the analysis, provided they had a pVL <50 c/mL and no toxicity at that visit, considering that this was a retrospective study and subjects were not tied up to an allocated treatment.

VF and factors associated with it were also pre-planned analyses. A secondary analysis assessed the percentage of patients remaining on the same regimen with a pVL < 50 copies/mL at the end of follow-up.

A relevant list of covariates was included in a multivariate Cox proportional model to determine factors independently associated with VF. The model was adjusted for age, intravenous drug use, hepatitis C co-infection, number of prior NNRTIs received, prior NNRTI treatment interruptions, presence of 3TC (versus FTC) in the regimen, inclusion of NVP in the last regimen, and duration of HIV-1 infection.

All variables with a significant association (p<0.05) in the univariate analysis were introduced into the multivariate model. The multivariate analysis was run in the overall cohort and also excluding subjects already on NVP at the time of the switch. The duration of treatment and time to VF were estimated using the Kaplan-Meier method. All statistical analyses were performed using SPSS software for Windows (version 15.0; SPSS Inc, Chicago, IL, USA).

## Results

### Baseline characteristics

We identified 367 patients having started a combination including NVP plus TDF plus FTC (or 3TC). Of them, 26 were treatment naïve when they initiated this combination, and were excluded. Cohort demographics of the remaining 341 are shown in Table 1. Most study subjects were male (72%), with a mean age of 42 years. The mean time with undetectable pVL at regimen initiation was 48 months, and had been exposed to a median number of 6 drugs. Prior VFs before initiating NVP were documented in 24% of them.

**Table 1:** Patient characteristics at the initiation of NVP plus TDF plus FTC (or 3TC) as a switch strategy (n = 341).

Age (yrs, mean [SD])	42.2 (8.7)
Gender (male, %)	246 (72)
Risk category for HIV acquisition (n, %)	
MSM	125 (37.3)
Intravenous Drug Users	92 (27.5)
Heterosexual	92 (27.5)
Other	32 (9.4)
Hepatitis B or C Co-infection (n, %)	
Hep C	98 (29.8)
Hep B	25 (7.7)
Pregnancy	7 (7.4)
Nadir CD4 cell count (cells, mean [SD])	239 (148)
Baseline CD4 cell count (cells, median [IQR])	492 (331,7)
Time of HIV-1 infection (months, mean [SD])	128 (69)
Time with undetectable viral load at regimen initiation	48 (33)
(months, mean [SD])	
Prior Antiretroviral exposure (n of drugs, median [IQR])	6 (4,8)
Number of prior NRTI	4 (2,5)
Number of prior NNRTI	1 (1,1)
Number of prior PI	1 (1,2)
Prior NRTI mono or dual therapy (n, %)	146 (43)
Both NRTI mono and dual NRTI prior therapy	60 (18)
Prior virologic failures documented (n, %)	79 (24)
Prior NNRTI documented treatment interruption (n, %)	99 (29.2)
NNRTI interruption only once	1 (23)
More than 1 NNRTI interruption	44 (13)
Drug previously interrupted:	
Nevirapine	60 (18)
Efavirenz	18 (5)
Viral load at baseline < 50 copies/mL (n, %) *	264 (78.3)
Lamivudine present in the last regimen	193 (57)
Emtricitabine present in the last regimen	66 (19)
Tenofovir present in the last regimen	126 (37)
Nevirapine present in the last regimen	173 (51)
Drug substituted by NVP	
Efavirenz	56 (33)
Protease inhibitor (indinavir, nelfinavir, saquinavir)	40 (24)
Boosted protease inhibitor (lopinavir, atazanavir, darunavir)	60 (36)
Other (raltegravir, etravirine)	12 (7)
Received 3TC + NVP + TDF	159 (47)
Received FTC + NVP + TDF	182 (53)

Data are median (IQR) or n (%).

* Some individuals in the early calendar years had an undetectable viral load at baseline, but with tests using at that moment a threshold of 80 or 200 copies/mL.

MSM: Men having sex with men; NRTI: Nucleos(t)ide reverse transcriptase inhibitor; NNRTI: non-nucleoside reverse transcriptase inhibitor; PI: Protease inhibitor.

### Reasons for initiation of the regimen

The main reasons for initiating the switch regimen were prior drug toxicity (169, 49.6%), treatment simplification (149, 43.7%), and pregnancy desire (5, 1.5%).

### Patient disposition at 48 weeks

Overall, 295/341 (86.5%) patients had a pVL <50 copies/mL at 48 weeks (mITT, S = F), and 10 (2.9%) experienced confirmed VF at 48 weeks. Drug toxicity led to treatment discontinuation in 22 (6.6%) subjects, and 14 (4.0%) experienced a voluntary treatment discontinuation.

Toxicity was specifically assessed in all NVP-naïve subjects at the initiation of the study regimen (168 out of 341). Of them, NVP was discontinued in 20 (11.9%), mainly due to early development (most of them at first trimester) of rash or laboratory liver abnormalities. Only 3 (2%) subjects developed grade 4 transaminase increases, none a severe clinical liver event, and none grade 4 rash.

In an on-treatment analysis, 96.2% of subjects receiving NVP at 48 weeks had a pVL <50 copies/mL.

### Patient disposition at the last follow-up visit

Patients stayed on the regimen for a median of 176 (57; 308) weeks and 215 (63.5%) patients discontinued the study regimen at any time during the follow-up. The reasons for treatment discontinuation at the last available control were: lost to follow-up (43, 12.6%), voluntary treatment interruption (37, 10.9%), recruitment for a randomized clinical trial (37, 10.9%), toxicity (34, 10.1%), confirmed VF (23, 6.7%; 17 on 3TC and 6 on FTC, p = 0.034), subsequent treatment simplification (18, 5.3%). Among individuals with immune discordance despite a suppressed viremia, 21 (6.1%) received proactive treatment changes (most of the latter empirically switched NVP to a PI/r, a common practice during some years). Therefore, only 57 (16.8%) subjects discontinued the treatment due to toxicity or lack of efficacy at 4 years.

**Fig 1:**
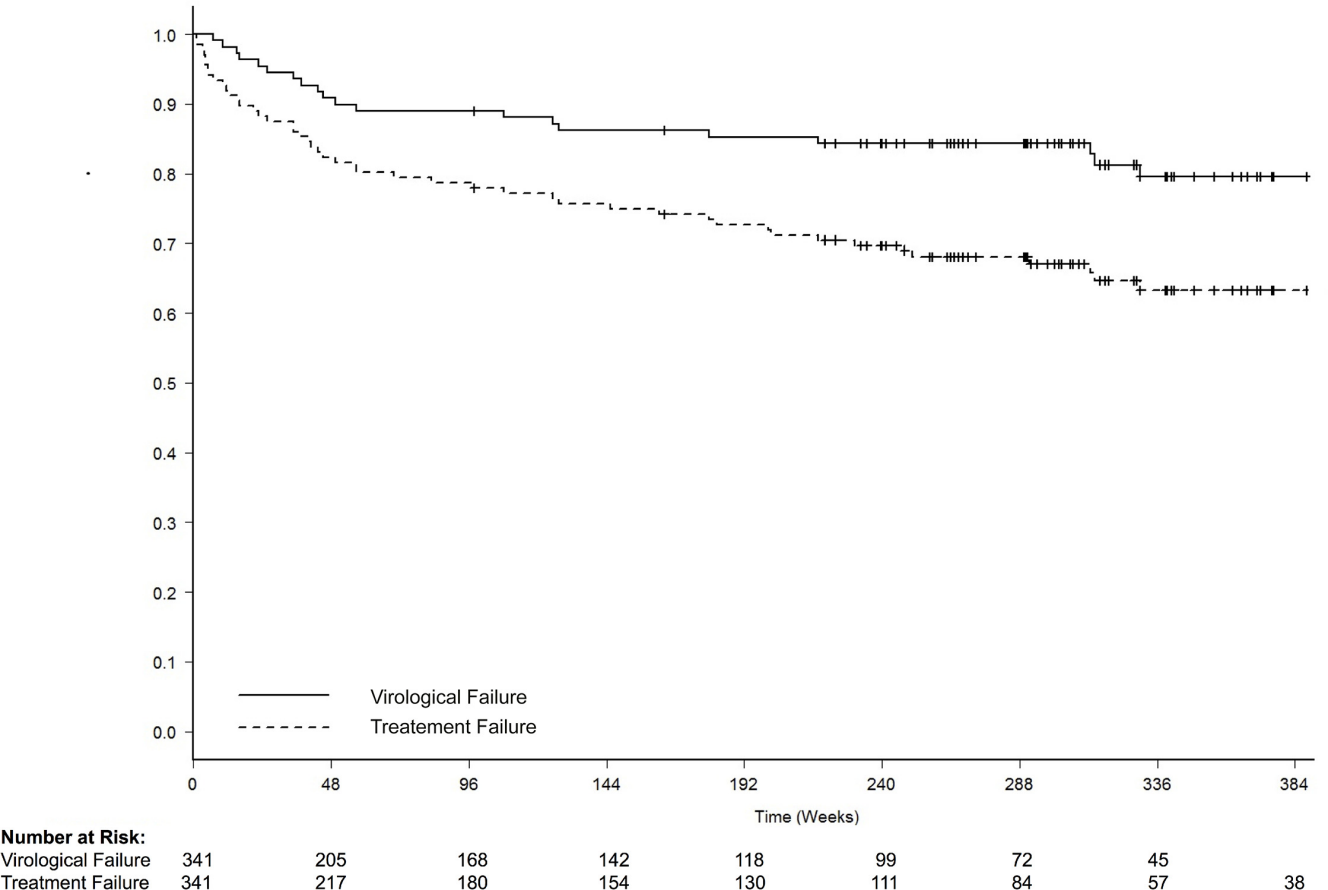
Time to virological failure and treatment failure through the long term follow-up. Virological failure was defined as two consecutive measurements of pVL >50 copies/mL. Treatment failure included subjects with virological failure, treatment discontinuations due to drug toxicity, and death.

Hence 156 (45.8%) patients discontinued the study regimen or follow-up in real clinical practice due to reasons unrelated to treatment efficacy. Of the overall cohort, 126 (37.3%) were still on the same regimen and with an HIV-RNA <50 copies/mL in their last control available (median 4 years). The median time to VF and treatment discontinuation are depicted in Fig 1.

### Factors associated with VF

In a multivariate analysis adjusted for variables described in Table 2, factors independently associated with VF were: intravenous drug use (HR 1.51; 95%CI 1.12, 2.04), longer time with undetectable pVL before regimen initiation (HR 0,98; 0.97, 0.99), number of prior NRTIs received (HR 1.49; 1.15, 1.93), number of prior NNRTIs received (HR 3.2; 1.6, 6.3), prior NVP interruptions (HR 1.54; 1.10, 2.17), prior efavirenz interruptions (HR 5.76; 1.11, 29.87), and NVP being present in the last regimen prior to current simplification (HR 0.57; 0.43, 0.76). Other factors significantly associated with VF in univariate analysis are shown in Table 2.

**Table 2:** Factors associated with virologic failure to a switch regimen composed of NVP plus TDF plus FTC (or 3TC) (n = 341).

	Univariate		Multivariate	
Variable	HR (95% CI)	p value	HR (95% CI)	p value
**Age**	0.99 (0.96, 1.03)	0.76		
**Gender**	1.41 (0.72, 2.78)	0.31		
**Intravenous Drug Users**	1.98 (1.09, 3.58)	0.02	1.51 (1.12, 2.04)	0.01
**Hepatitis B or C Co-infection**				
Hep C	1.58 (0.87, 2.87)	0.13		
Hep B	0.90 (0.32, 2.52)	0.85		
**Nadir CD4 cell count**	0.98 (0.79, 1.22)	0.86		
**Baseline CD4 cell count**	0.80 (0.69, 0.92)	0.00		
**Time of HIV-1 infection**	1.00 (1.00, 1.00)	0.92		
**Longer time with undetectable VL**	0.98 (0.97, 1.00)	0.01	0.98 (0.97, 0.99)	0.01
**Prior Antiretroviral exposure**	1.01 (0.89, 1.13)	0.93		
number of prior NRTI	1.18 (0.97, 1.43)	0.10	1.49 (1.15, 1.93)	0.00
number of prior NNRTI	2.38 (1.49, 3.85)	0.00	3.22 (1.64, 6.25)	0.00
number of prior PI	1.03 (0.81, 1.31)	0.82		
**Prior NRTI mono and dual therapy**	1.14 (0.65, 2.01)	0.65		
**Prior VF documented**	1.76 (0.96, 3.25)	0.07		
**Prior NNRTI treatment interruption**	2.13 (1.20, 3.78)	0.01		
Nevirapine interruptions	1.72 (0.86, 3.45)	0.13	1.54 (1.10, 2.17)	0.01
Efavirenz interruptions	3.11 (1.23, 7.90)	0.02	5.76 (1.11, 29.87)	0.04
**VL at baseline <50 c/mL** *	0.22 (0.12, 0.39)	0.00		
**3TC present in the last regimen**	0.72 (0.40, 1.30)	0.27		
**FTC present in the last regimen**	0.30 (0.04, 2.18)	0.23		
**TDF present in the last regimen**	1.01 (0.53, 1.95)	0.97		
**NVP present in the last regimen**	0.32 (0.18, 0.57)	0.00	0.57 (0.43, 0.76)	0.00
**3TC (vs FTC) in the regimen**	2.48 (1.38, 4.46)	0.00		

* Some individuals in early calendar years had an undetectable viral load at baseline, but with tests using a threshold of 80 or 200 copies/mL.

VL: viral load; VF: virologic failures; NNRTI: non-nucleoside reverse transcriptase inhibitor; NRTI: nucleoside analogue reverse transcriptase inhibitor; PI: protease inhibitor.

### Resistance selection at failure

Population genotypes at failure were available in all 23 subjects (6.7%) experiencing confirmed VF (Table 3). None of them had previous resistance tests available. Wild-type HIV-1 was seen in 5 (22%) of them. Sixteen out of 23 (70%) had NNRTI mutations (Y181C/I/D: 10; K103N: 6; V106A/I: 3; Y188C/L: 2; K101Q/E: 2; M230L: 1; P225H: 1; A98G: 1; V108I: 1; F227L: 1; K238T: 1), and 10 had >1 NNRTI mutation.

**Table 3:** Mutations shown at failure in the reverse transcriptase and protease, and NRTI included in the regimen together with NVP and TDF (3TC vs FTC).

3TC/FTC	Reverse transcriptase	Protease
FTC	A62V,K65R,Y181C,M184V	None
FTC	A62V, K65R, V75I, K103N, Y181C, M184V, M230L,	L63P
3TC	A62V,L74V,K103N,V106A,M184V,T215S,P225H	L63P
3TC	A62V, K65R, K101Q, Y181C	K20M, M36I, M46I, Q58E, L63P, L90M, I93L
3TC	V118I, M184V, Y188C	L63P
FTC	M41L, E44D, D67N, K70R, M184V, T215Y, K219Q	L63P
3TC	Y181I, M184V	G16E
3TC	M41L,A62V,T69N,K70R,K103N,V108I,M184V,T215F,K219E	R41K, L63P, A71V, V77I, L90M, I93L
3TC	D67N, K103N, Y181C, M184V, K219E	I62V, I64V
3TC	None	L63P
3TC	A62V, K65R, A98G, Y181C, M184V	L63P
3TC	K65R, M184V, Y188L	I15V,V77I, I93L
FTC	None (wild-type)	V77I
FTC	L74V/L, Q102K/R, K103K/N, D177E/G, Y181Y/D, M184V, G190G/A	K20R, M36I, L63P
3TC	K65R, V108I, Y181C, M184V	13V, K20T, V32I, E35D, M36I, K43T, M46I, I47V, F53L, L63P, I66F, A71V, G73S, V77I, L90M,
3TC	M41L, E44D, D67N, T69D, V118I, Y181C, M184V, G190A, L210W, T215Y, V106V/I, F227F/I	L33V
3TC	None	M36I,L63P,V77I
3TC	K103N/S,Y181C,M184V	L33V, R41K, I64V, I13V/I, L63P
FTC	K101E, G190A, K238T	L10V, I13V, L63P
3TC	None	I13V, M36I, L63P
3TC	M41L, M184V, L210W, T215Y	V118I
3TC	None	I13V, L63P, V77I
3TC	K65R, V106A, M184V	None

M184V was selected in 16 (70%) subjects: 12 treated with 3TC and 4 with FTC (p = 0.04). Seven patients had K65R (6 associated to M184V, none with thymidine-analogue mutations [TAMs]), 5 of them treated with 3TC and 2 with FTC. Six patients selected A62V, with K65R selected in 4 of them, and none with Q151M or T69 insertions. Five patients harboured TAMs. Three subjects harboured major protease mutations (V32I, M46I, I47V, L90M), selected in prior failures.

## Discussion

The 48-week (2.9%) and long-term (6.7% at 4 years) rates of VF of a switch regimen composed of NVP plus TDF plus FTC (or 3TC) in real clinical practice are low, and similar to those of other common switch strategies seen in similar cohorts and recommended in international guidelines [1;2;27;28]. These strategies include unboosted atazanavir, rilpivirine, raltegravir or elvitegravir/cobicistat, and the corresponding rates of VF in their pivotal clinical trials were 1–8% [7;29;30]. However, data from prospective clinical trials are not comparable to those of cohorts including patients seen in everyday circumstances, and the rates of VF reported in cohorts have been higher and similar to our series [5;7;28–32]. Actually, raltegravir showed higher rates of VF in a randomized switch clinical trial (9.1% at 24 weeks), early terminated by a DSMB because of lower than expected virological efficacy [33;34].

Whilst VF was infrequent, drug resistance mutations against NNRTIs and NRTIs were frequently isolated in patients with VF, in agreement with what has been seen in pivotal trials with NVP in naives, and also with other drugs with a similarly low genetic barrier to resistance (efavirenz, rilpivirine, raltegravir and elvitegravir/cobicistat) in initial therapy [31;33–42]. On the other hand, unboosted atazanavir maintains a high genetic barrier to resistance in switch despite the absence of pharmacokinetic boosting, and no major protease mutations isolated in failures in randomised clinical trials even in the long term, albeit they have indeed been reported in some cohorts in real clinical practice [28;29].

Reassuringly, the frequency and type of mutations seen in our series was concordant with what has been previously seen in other NVP studies, with a potential impact of some of them against the activity of etravirine in subsequent treatments [43]. The most common emergent NNRTI mutations were Y181C/I/D, K103N and V106A/I [44].

The rate of K65R selection was low was, but appeared in approximately one of every three failures, usually with M184V, and more frequently in those treated with 3TC (vs FTC) [35;36].

We observed a significantly higher rate of VF in individuals treated with 3TC instead of FTC, with a significantly higher rate of selection of M184V as well. Nevertheless, these data must be interpreted with caution, as calendar years when subjects received 3TC or FTC were different—as well as the number of pills in the regimen—and unmeasured confounders could exist. However, this is concordant with a double risk of VF (adjusted OR 2.2; 95%CI 1.1, 4.6) for 3TC vs FTC found in treatment-naives [26;45]. Some previous reports have suggested potential differing resistance profiles for FTC and 3TC when administered in combination with TDF [46;47]. Moreover, previous reports have shown differing resistance profiles for FTC and 3TC when administered in combination with TDF [46;47]. Therefore, our findings suggest caution against substituting FTC for 3TC, at least in NVP- and TDF-based regimens, while other series review their data [1]. A shorter intracellular t_1/2_ of activated triphosphate 3TC compared to FTC (15 h versus >39 h), a 4- to 10-fold lower antiviral potency of FTC in a range of cell line *in vitro* passages, and the ability of FTC to inhibit cellular efflux proteins such as the multidrug resistance–associated proteins could account for a lower forgiveness of 3TC, at least when combined with NVP [23;47–49]. This is due to the ability of FTC to inhibit the activity of the cellular efflux proteins, such as the multidrug resistance–associated proteins, that extrude the drugs out of the CD4+ cells [47].

No subjects selected NRTI resistance mutations without NNRTI ones, thus confirming that NNRTI resistance is selected first, in agreement with previous reports [50;51].

In an adjusted multivariate analysis, we found an independent association of VF with intravenous drug use, time with undetectable VL, number of prior NRTIs or NNRTIs received, and prior NVP or efavirenz interruptions. Actually, intravenous drug users and higher antiretroviral drug exposure are variables universally associated to increased rates of VF to any switch regimen [10;34;52]. The long half-life of NNRTIs, as compared with that of some NRTIs, may allow a long terminal tail in plasma pharmacokinetics with suboptimal late NNRTI functional monotherapy in unplanned treatment interruptions. In addition, repeated drug holidays (>48 h of drug cessation) have been previously associated with VF to NVP and efavirenz [52]. These findings have clinical translation indeed. Some studies using standard and ultrasensitive techniques have been able to detect NNRTI-associated mutations in up to 14–16% of individuals who discontinued a NNRTI-based regimen with a pVL <50 copies/mL, particularly with a simultaneous interruption (instead of a staggered interruption) of all drugs in the regimen [53;54]. Moreover, these interruptions led to a 14-fold increased risk of detecting genotypically resistant HIV-1-RNA in female genital tract secretions, therefore potentially increasing the risk of HIV transmission [55].

Therefore, reinitiation of NVP plus TDF plus FTC (or 3TC) should be discouraged in subjects experiencing unplanned treatment interruptions, even with an undetectable pVL at the time of treatment withdrawal.

The main reasons for initiating the regimen in our series were prior toxicity and treatment simplification, which still remain as the main reasons currently.

The rates of treatment discontinuation due to adverse events in our cohort (6.6% at 48 weeks, 10.6% overall at 4 years, and 11.9% in those initiating new NVP) are concordant with rates seen in other NVP or efavirenz studies [10;12;35;36], but are higher than those observed with some other switch strategies and constitute the main limitation of this regimen [29;30;33;34]. This is a drug-related effect of NVP, and suspicion of hypersensitivity reactions or increases in liver transaminases were the most frequent reasons for stopping the regimen, mainly during the first 12 weeks. These early toxicity-associated withdrawals prevented the demonstration of non-inferiority versus efavirenz in initial treatment in the 2NN study as well as in a recent systematic review [56;57]. Grade 4 adverse events were seen in only 2% of the patients and no severe clinical events were reported among the 341 subjects. Not unexpectedly, those receiving NVP in their baseline regimen before the switch were indeed less prone to toxicity. The greater risk of symptomatic hepatic or skin events, including serious and potentially life-threatening events, although the latter not observed in our series, may remain an intrinsic restriction to new NVP initiation in the future, as compared to the lower intrinsic toxicity of newest drugs.

However, the most frequent reasons for withdrawing the regimen in real clinical practice were not related to toxicity or lack of efficacy, but proactive treatment changes, recruitment for clinical trials, or patients being lost to follow-up, with subjects having suppressed viremia at that time point.

Generic substitution is one mechanism of curtailing prescription drug expenditures. Currently available as a generic and with reference pricing, a cost/efficacy assessment done by the Spanish GESIDA Society has shown that NVP plus TDF/FTC (or 3TC) constitutes a cost-effective treatment in Europe despite the availability of many new regimens [58]. It is administered once-daily as a two-pill regimen, and is commonly used in developing countries as well as developed countries with economic constraints [58].

These findings inform regimen management in clinical practice, and would support the long-term maintenance of this strategy in subjects without initial toxicity. Actually, newer antiretroviral drugs have not demonstrated advantages in switch studies in subjects treated with NVP plus TDF/FTC [7].

Our study is subject to the limitation of its retrospective design, which could lead to bias with unmeasured confounding factors such as treatment adherence or channelling prescription by physicians. We specifically made every effort to capture all subjects receiving the first dose of the regimen, to avoid underestimation of toxicity. The study included subjects who changed the whole regimen and subjects who were already receiving NVP and only changed the NRTI backbone. However, both subgroups have been analysed separately in a sensitivity analysis to pinpoint the toxicity of NVP. Nonetheless, the study reports the largest cohort of patients treated with this switch regimen so far, and the results are consistent and robust through adjusted and sensitivity analyses. Important information gleaned from the study includes higher risk of failure with such a switch in a setting for subjects who had previously been exposed to NNRTI and had a history of treatment interruption.

In conclusion, a simplification regimen with NVP plus TDF and FTC (or 3TC) in pre-treated subjects maintained virologic suppression with a low risk of short and long-term treatment or VF in subjects without prior NNRTI treatment interruptions, and a low rate of long-term adverse events. The rates of VF are similar to other switch strategies, and no unexpected mutations or patterns of mutations have been selected at failure. These findings do not suggest increased early or late VF rates with this regimen when used as a simplification strategy. However, the rates of discontinuation due to early toxicity were higher. While potentially severe initial toxicity might limit its new initiation in the future, these data support caution against a systematic proactive switch of those subjects successfully treated with this regimen towards newest drugs until clinical advantages to patients are demonstrated in randomised clinical trials.
